# Protective Effects of Scutellarin on Type II Diabetes Mellitus-Induced Testicular Damages Related to Reactive Oxygen Species/Bcl-2/Bax and Reactive Oxygen Species/Microcirculation/Staving Pathway in Diabetic Rat

**DOI:** 10.1155/2015/252530

**Published:** 2015-03-12

**Authors:** Lingli Long, Jingnan Wang, Xiaofang Lu, Yuxia Xu, Shuhui Zheng, Canqiao Luo, Yubin Li

**Affiliations:** ^1^Translation Medicine Center, The First Affiliated Hospital, Sun Yat-Sen University, 58 Zhongshan 2nd Road, Guangzhou 510080, China; ^2^Department of Pathology, The First Affiliated Hospital, Sun Yat-Sen University, 58 Zhongshan 2nd Road, Guangzhou 510080, China; ^3^The Reproductive Center of the First Affiliated Hospital, Sun Yat-Sen University, 58 Zhongshan 2nd Road, Guangzhou 510080, China

## Abstract

The goal of our study is to evaluate the effect of Scutellarin on type II diabetes-induced testicular disorder and show the mechanism of Scutellarin's action. We used streptozotocin and high-fat diet to establish type II diabetic rat model. TUNEL and haematoxylin and eosin staining were used to evaluate the testicular apoptotic cells and morphologic changes. Immunohistochemical staining was used to measure the expression level of vascular endothelial growth factor and blood vessel density in testes. Oxidative stress in testes and epididymis was tested by fluorescence spectrophotometer and ELISA. The expression of Bcl-2/Bax and blood flow rate in testicular vessels were measured by western blot and Doppler. Our results for the first time showed that hyperglycemia induced apoptotic cells and morphologic impairments in testes of rats, while administration of Scutellarin can significantly inhibit these damages. This effect of Scutellarin is controlled by two apoptotic triggers: ROS/Bcl-2/Bax and ROS/microcirculation/starving pathway.

## 1. Introduction

Diabetes mellitus (DM) is one of the common metabolic diseases characterized by hyperglycemia. Global prevalence of DM was approximately 2.8% in 2000 and is estimated to reach 4.4% in 2030 [1]. Sustained hyperglycemia can lead to complications in multiple organs, such as neuropathy, retinopathy, nephropathy, male impotency, and cardiovascular diseases [2]. Several studies from experimental animals and the diabetic men demonstrated that sustained hyperglycemia results in reproductive complications, as high glucose possibly lead to oxidative stress and cell apoptosis, which result in structure and functions impairments and finally contribute to infertility [3]. Diabetes has been recently diagnosed in younger persons and the age barrier has been broken. Therefore, diabetes-induced reproductive dysfunction is emerging as a new and urgent challenge [4].

Increased oxidative stress in diabetes has been considered to contribute to the damage of reproductive system [5]. Oxidative stress has been used to describe the disturbance between the production of reactive oxygen species (ROS) and the ability to detoxify reactive intermediates in biological system [6]. Imbalance in the redox state of cells can increase the level of free radicals and peroxides which can impair the components of the cell, such as protein and DNA, and result in toxic effects [7]. Recent study showed that increased oxidative stress in diabetic rats induced damage of testicular DNA, depletion of sperm cells, and delay of spermatogenesis, resulting in infertility [8].

DM leads to vascular complications, associating with the appearance of macroangiopathy, microangiopathy, and diabetic thrombophilia in multiple organs [9]. Actually, studies have demonstrated that diabetes can bring about oxidative stress-induced microvascular complications [10]. Vascular endothelial growth factor (VEGF), a signal protein that controls angiogenesis and vasculogenesis, plays an important role in diabetes-induced vasculopathy [11]. During the processes of diabetes, dyslipidemia, hypertension, and damage of NO synthesis occurred and decreased the blood flow rate in the vessels, which can result in tissue hypoxia and unbalance expression of VEGF [12], whereas little was known about the change of testicular microcirculation and the role of VEGF in diabetes.

Currently, insulin and oral hypoglycemic agents are commonly used for diabetes. Although big effort has been made for controlling the blood glucose, the complication of diabetes is still the major reason to cause organ dysfunction and death [13]. Thus, novel regimens are still needed to improve complications of diabetes. The effect of agents especially on alleviating reproductive complications in diabetes has been rarely discussed.

**Scheme 1:**
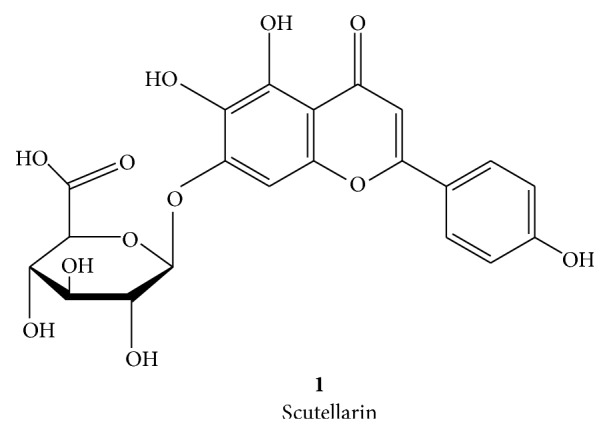


Recent studies show that many natural products have antidiabetic activity [14] and significant antioxidant capacity [15]. Some of the natural products have been used as alternative and complementary medicine for diabetes [16]. Scutellarin (40,5,6-trihydroxyflavone-7-glucuronide, linear formula: C21H18O12, SCU) extracted from* Erigeron breviscapus* (Vant.) is one of the flavone glycosides. Hand.-Mazz. SCU Scheme 1 possesses antioxidant property and has been proved to improve microcirculation [17]. Preclinical data showed that SCU can ameliorate diabetic nephropathy through reducing ROS generation [18] and reduce cardiomyocyte apoptosis by upgrading the expression of proapoptotic genes in Bcl-2 family [19]. Acute and toxicological study showed that LD_50_ value of SCU could not be measured, and its maximum tolerance was 10 g/kg. Continuous oral intake of SCU (100 and 500 mg/kg) up to one month did not lead to any death or organ damage in rats [20]. Because of its satisfactory results, safety, and tolerance, SCU has been widely utilized in treatment of diseases associated with diabetes [21]. In this study, we first evaluated the effect of SCU on improving testicular dysfunction and structure and studied the mechanisms of its actions using diabetic model in rat.

## 2. Material and Method

### 2.1. Experimental Animal

All animal protocols were approved by China Council on Animal care and Sun Yet-San University Committee and have therefore been performed in accordance with the ethical standards laid down in the 1964 Declaration of Helsinki and its later amendments. Adult male Wistar rats (200 g–250 g; 6 weeks of age) were purchased from animal facility of Sun Yat-Sen University and housed in stainless steel cages. Rats were randomly divided into three groups: control group (*n* = 8), diabetes group (*n* = 8), and diabetes + SCU group (*n* = 8). Rats were fed under controlled environment at 25 ± 3°C, humidity of 40–65% and 12 h light/dark cycle, and free access to food and drink water. Normal food (Animal Facility of Sun Yat-Sen University, Guangzhou, China) was given to the control group. High-fat diet (HFD, Guangdong Medical Laboratory Animal Center, Guangzhou, China), containing 30% fat, 15% protein, and 55% carbohydrate, was used in diabetes group and diabetes + SCU group to induce insulin resistance. At week 8, a single dose of streptozotocin (STZ, Sigma-Aldrich, MO, USA, S0130; 65 mg/Kg) dissolved in 0.1 M citrate-phosphate buffer was freshly prepared and intraperitoneally administered into diabetes group and diabetes + SCU group to induce the type II diabetes mellitus (HFD feeding was continued). The same volume of 0.1 M citrate-phosphate buffer was used as vehicle control for control group. The rats with blood glucose levels higher than 13 mmol/L for three times were considered as type II diabetes mellitus (T2DM). Blood obtained from rat tail-vein was evaluated to determine the levels of blood glucose using automated blood glucose analyzer (Roche, Model GC, Switzerland). After one week of the injection of STZ, rats in diabetes + SCU group were intragastrically administrated with SCU (purity > 98.5%, Yunnan Plant Pharmaceutical, Yunnan, China; 100 mg/kg/day) dissolved in a phosphate buffer (0.1 M, pH 7.3 at 20 mg/mL) every day for 2 months. The dose of STZ and SCU was according to previous published work [17, 22]. The same volume of phosphate buffer was used as vehicle control for control group and diabetes group. At week 16, all rats were sacrificed with injection of chloral hydrate as anesthetic, and then testes and epididymis were removed and weighed.

### 2.2. TUNEL Staining to Evaluate Apoptotic Cells

Apoptotic cells in testicular were measured through TUNEL assay with the In Situ Cell Death Detection Kit, POD (Roche, Basel, Switzerland) following the manufacturer's instructions. Testicular tissue was fixed in 4% paraformaldehyde, embedded in paraffin, and then sectioned at 5 *μ*m. Briefly, slides were dewaxed and rehydrated in xylene and ethanol followed by incubation with proteinase K working solution at 37°C for 20 min. After rinsing with PBS, the samples reacted with TUNEL reaction mixture (50 *μ*L) at 37°C for 1 hour followed by rinsing the slides with phosphate-buffered saline to stop the reaction. Apoptotic cells were analyzed by randomly counting the TUNEL positive cells from sixty cross-sections of seminiferous tubule/slide under the fluorescence microscope at ×200 and ×400 magnification (Olympus, TH4-200, Tokyo, Japan). Results were quantitative analysis as TUNEL positive cells per 100 cells (Image-Pro Plus 6.0).

### 2.3. Haematoxylin and Eosin Staining

Five sections from each group were cut consecutively from paraffin-embedded block. After dewaxing in xylene and dehydrating in graded concentrations of alcohol, sections were stained by haematoxylin and eosin (H&E) used to evaluate testicular morphology. The results were observed under light microscope at ×200 magnification.

### 2.4. Immunohistochemical Studies

Immunohistochemistry was performed according to Sisman et al. [23] and the manufacturer's instructions. The slides were prepared as described in TUNEL assay, immersed in citrate buffer, and heated in a microwave oven for 15 min. Endogenous peroxidase was blocked with 3% H_2_O_2_ solution in methanol for 20 min. After three times washing with TBS, the slides reacted with anti-VEGF primary antibody (Abcam, Britain, dilution 1 : 200) or anti-vWF primary antibody (Abcam, Britain, dilution 1 : 200) and then reacted with anti-rabbit secondary antibody at 37°C for 30 mins followed by three washes with PBS, respectively. Diaminobenzidine (DAB Kit; Beijing Zhongshan Biotechnology Co., China) was used to visualize immunoreactive proteins. The protein levels were detected using Polink-2 Plus IHC Detection System (Beijing Zhongshan Biotechnology Co., Beijing, China) after staining with diaminobenzidine (DAB Kit; Beijing Zhongshan Biotechnology Co., China) to visualize immunoreactive proteins following the manufacture instruction. Diaminobenzidine (DAB) was added to the slides for 3 min as a chromogen. Followed by rinsing in running tap water, slides were counterstained with hematoxylin (blue). Finally, slides were dehydrated in absolute ethanol and mounted. All of them were observed under the light microscope at ×200 magnification and ×400 magnification (Olympus, Japan).

### 2.5. Evaluation Testicular Concentrations of ROS and Malondialdehyde (MDA)

ROS were determined following the method of Driver et al. [24]. Testes were taken from rats under ethylether anaesthesia and were grinded by Tube Mill control (IKA, Germany). We diluted homogenates to 5 mg tissue/mL in ice-cold Locke's buffer and then transferred samples into 24-well plates (0.45 mL/well). All the samples were incubated at room temperature for 10 min. After that, 5 *μ*L of DCFH-DA (10 *μ*M final concentration) was dropped into each sample. During 15 min incubation at room temperature, DCFH-DA can incorporate into any membrane-bound vesicles and esterases can cleave the diacetate group. At that time, 40 *μ*L of the Fe^2+^ was put into the samples, which can transform the DCFH to fluorescent product DCF measured by fluorescence spectrophotometer (F-7000, Hitachi, Japan) with excitation at 485 nm and emission at 530 nm.

The concentration of MDA was considered as a marker of lipid peroxidation, which can reveal the oxidative damage as a result of diabetes. Testes were removed from rats under ethylether anaesthesia and were grinded by Tube Mill control (IKA, Germany). Homogenates were centrifuged at 4°C, 10000 rpm for 10 min. The supernatant was pipetted into 96-well plates and used for the ELISA test. The whole blood samples were taken from the abdominal vein of rats, and then they were spun to get serum. We detected the MAD level of testes and serum by Lipid Peroxidation (MDA) Assay Kit (Abcam, ab118970; Cambridge, UK), following the manufacturer's instructions.

### 2.6. Doppler Measurement of Blood Flow in Testicular Vessels

After anaesthesia by chloral hydrate, the testicular vasculature was localized with a MS 400 probe (18–38 MHz) for color Doppler sonography (VisualSonics Vevo 2100, Toronto, Canada) and Doppler waveforms were measured. The control steps were carried out following the manufacturer's instructions and previous published paper [25]. The red color indicated blood flow towards the transducer and the blue color indicated blood flow away from the transducer. Color images were shown in real time and Doppler spectral analyses were done. At least three measurements from each recording were carried out and the mean of the three readings was taken as the representative. 

### 2.7. Western Blot Analysis

Testis samples were immersed in lysed buffer and centrifuged at 13,000 rpm for 15 min at 4°C. Bicinchoninic Acid (BCA) Kit (Pierce, Rockford, IL, USA) was used to measure the protein concentration in the supernatants with steps according to the manufacturer's protocol. Total protein (50 *μ*g) was added to each lane onto 10% SDS-polyacrylamide gels. After electrophoresis, we used Tris-buffered saline containing 0.1% Tween-20 to wash the polyvinylidene fluoride (PVDF) membranes (Millipore, Billerica, MA, USA) and then incubated them with primary antibody: anti-Bcl-2 (diluted 1 : 1000, Cell Signaling Technology, Beverly, MA, USA), anti-Bax (diluted 1 : 1000, Cell Signaling Technology, Beverly, MA, USA), anti-vWF (diluted 1 : 1000, Abcam, Britain), and anti-VEGF (diluted 1 : 1000, Abcam, Britain) at 4°C overnight. Secondary antibody (1 : 5000, Pierce, Rockford, IL, USA) was added to the membrane for 2-hour incubation. Protein was visualized with Immobilon Western Chemiluminescent HRP Substrate (Millipore, Millipore Corp., Bedford, USA). Band intensity was quantified by densitometry using the Image J software (National Institutes of Health, Bethesda, MD, USA).

### 2.8. Blood Sampling and Serum Lipid Level Parameters Analysis

Blood samples (5 mL) were taken from the inferior vena cava before sacrifice at the week 16. Serum was spun at 1500 rpm for 30 minutes and stored at −20°C until used. These sera were sent to clinical laboratory of the First Affiliated Hospital, Sun Yat-Sen University, analyzed for triglyceride (TG), total cholesterol (TC), high density lipoprotein (HDL), and low density lipoprotein (LDL).

### 2.9. Statistical Analysis

The data were shown as the mean ± standard deviation. Significant differences between the groups were determined using a one-way ANOVA through the SPSS 19.0 software. *P* < 0.05 was considered statistically significant.

## 3. Results

### 3.1. General Features in Experiment Rats

**Figure 1:**
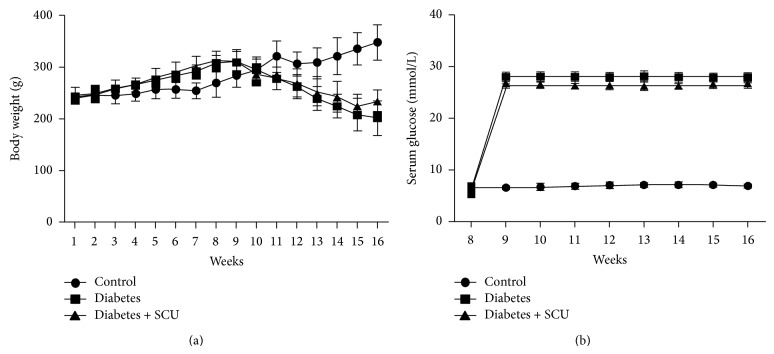
Body weights and serum glucose concentrations. STZ were administrated into diabetes group and diabetes + SCU group at week 8. One week later, diabetes + SCU group was daily administrated with SCU from week 9 to week 16. (a) Body weight and (b) glucose concentration were measured as described in methods.

Body weights and serum glucose of experimental rats were showed in Figure 1. Body weights in all groups were increased until administration of STZ. After injection of STZ, body weights of diabetes group and diabetes + SCU group showed a decreased tendency. Diabetes + SCU group daily receiving SCU showed a slower decreased tendency. Body weights in control group were kept on growing. Serum glucose suddenly exceeded the normal level in diabetes group and diabetes + SCU group after STZ injection (Figure 1(b)). Serum glucose in diabetes + SCU group was lower than diabetes group, but there were no statistically significant differences between them (*P* > 0.05). The data of body weight and serum glucose from week 1 to week 16 were present as mean ± SEM (see Supplemental Table 1 of the Supplementary Material available online at  http://dx.doi.org/10.1155/2015/252530).

The testicular weights and epididymal weights of rats were presented in Table 1. Comparing with the control group, decreased testicular weights and epididymal weights were shown in the diabetes group (*P* ≤ 0.05). In the diabetes + SCU group, both of these weights were heavier than those of diabetes group (*P* ≤ 0.05).

### 3.2. Oxidative Impairments in Testes of Diabetic Rats

**Figure 2:**
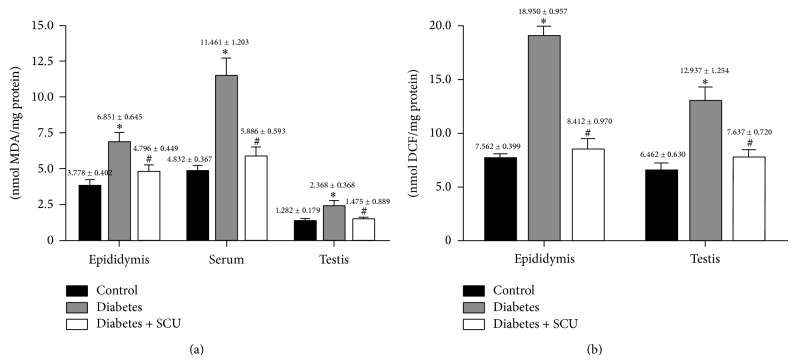
Oxidative stress measured as the concentration of MDA and ROS. MDA was measured to determine lipid peroxidation levels of testes, epididymis, and serum (a) and fluorescent product DCF was measured to determine ROS levels of testes and epididymis (b). Data in (a) and (b) are presented as means ± SEM (*n* = 8); ^*^ *P* ≤ 0.05, compared to control group; ^#^ *P* ≤ 0.05, compared to diabetes group.

MDA and ROS concentrations in testes, epididymis, and serum were shown in Figure 2. Compared with control, obviously higher concentrations of MDA and ROS in testes, epididymis, and serum were shown in diabetes group (*P* < 0.05). The increased MDA and ROS can be effectively suppressed by SCU, while concentrations of MDA and ROS have significant difference between diabetes group and diabetes + SCU group (*P* < 0.05).

### 3.3. SCU Treatment Reduced Testicular Apoptosis in Diabetic Rats

**Figure 3:**
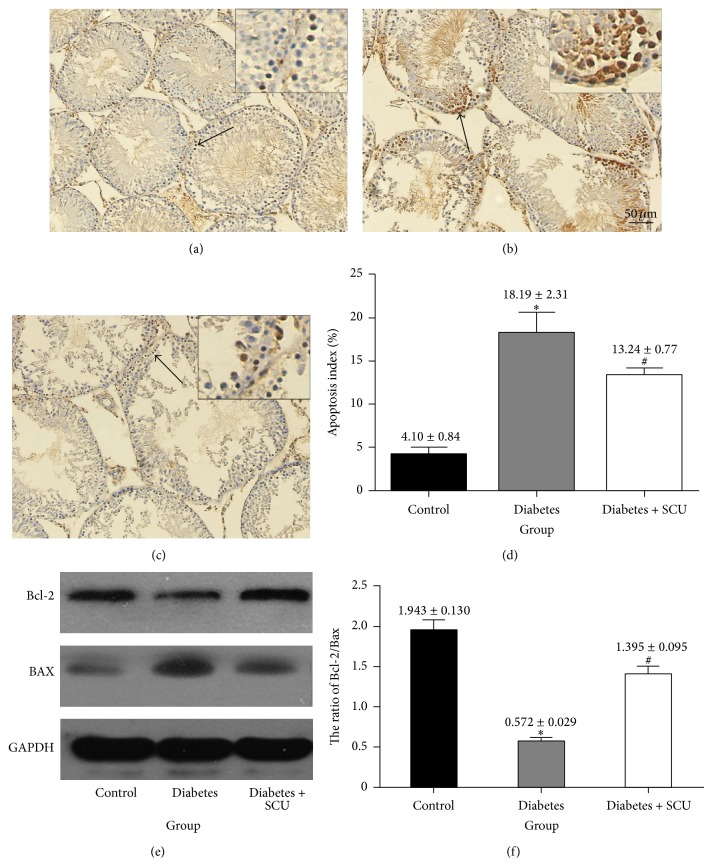
Diabetes induces ROS production and lipid peroxidation with enhanced cellular apoptosis. (a)–(d) TUNEL staining of testes sections from control, diabetes, and diabetes + SCU rats. TUNEL-positive cells (indicated by arrows) in representative images are shown in (a), (b), and (c) (200x magnification and 400x magnification). Hoechst was used as cell nucleus staining. The % of apoptotic cells were quantified in (d) and calculated following the formula Apoptosis index = apoptotic cells/(apoptotic cells + normal cells) (d). Data in (d) are presented as means ± SEM, 3 fields per section and 5 sections from each testis. *n* = 8 rat for each group, and analyzed by *t*-test. ^*^Significantly different from control group (*P* ≤ 0.05); ^#^significantly different from diabetes group (*P* ≤ 0.05). (e) Western blot analysis was used to determine the protein expression of Bcl-2 and Bax in testicular tissue. GAPDH was used as loading control. (f) The ratio of Bcl-2/Bax was determined by quantitative evaluation of the protein expression of Bcl-2 and Bax using densitometry analyses. Samples were from three individuals in each group. Data in (f) are presented as mean ± SEM. ^*^Significantly different from control group (*P* ≤ 0.05); ^#^significantly different from diabetes group (*P* ≤ 0.05).

An obvious increasing of testicular apoptosis in diabetic rats at 2 months after diabetes onset, measured by TUNEL staining (Figures 3(a), 3(b), and 3(c)), was found along with a significant increase in Bcl-2/Bax ratio (Figure 3(e)). In the control group, only a few apoptotic cells were present in testis (Figure 3(a)), but they obviously increased in diabetic group (Figure 3(b)). SCU treatment resulted in decrease of TUNEL-positive cells (Figure 3(c)), though it was still larger than control group (*P* < 0.05; Figure 3(c)). The apoptosis index was 3.7%, 21.9%, and 11.8% in control group, diabetes group, and diabetes + SCU group, respectively (Figure 3(d)). We also used western blot to confirm the antiapoptosis effects of SCU on testes of diabetic rats (Figure 3(e)). Rats in diabetes group showed a decreased expression of Bcl-2 and increased expression of BAX, while SCU treatment led to increased expression of Bcl-2 and decreased expression of BAX (Figure 3(e) with quantifications of ratio of Bcl-2/BAX in Figure 3(f)). These data are consistent with our TUNEL results that diabetes induced testicular apoptosis, which can be alleviated by SCU treatment.

### 3.4. Changes of Testicular Morphology

**Figure 4:**
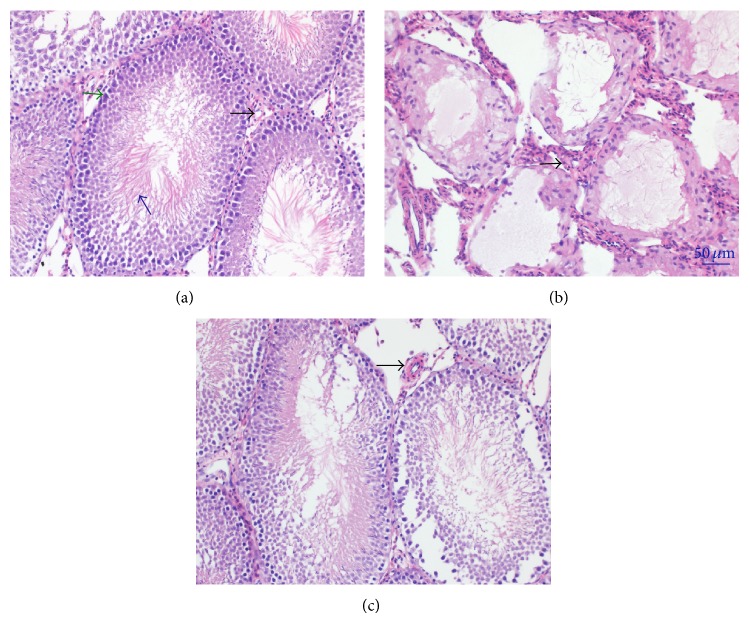
H&E staining of testicular tissue changes. (a) H&E staining in control rats (200x), germ cells (green arrow), spermatozoa (blue arrow), and blood vessel (black arrow) was indicated. (b) H&E staining in diabetic rat (200x). (c) H&E staining in diabetes + SCU rat (200x).

Control group showed the normal testicular structure and regular seminiferous tubular morphology with plenty germ cells and spermatozoa. The blood vessel walls in the control group were thin and smooth (Figure 4(a)). In the diabetes group, atrophic seminiferous tubules with few germ cells and abnormal spermatozoa were detected. The structures of seminiferous tubules were disrupted. Also the thickness of blood vessel walls were significantly increased (Figure 4(b)). Treatment with SCU effectively increased the number of germ cells and spermatozoa, but germ cells were still disordered with some sloughing. Blood vessel walls were thinner than those in diabetes group (Figure 4(c)).

### 3.5. SCU Treatment Protects against Microcirculatory Injury in Diabetic Rats

**Figure 5:**
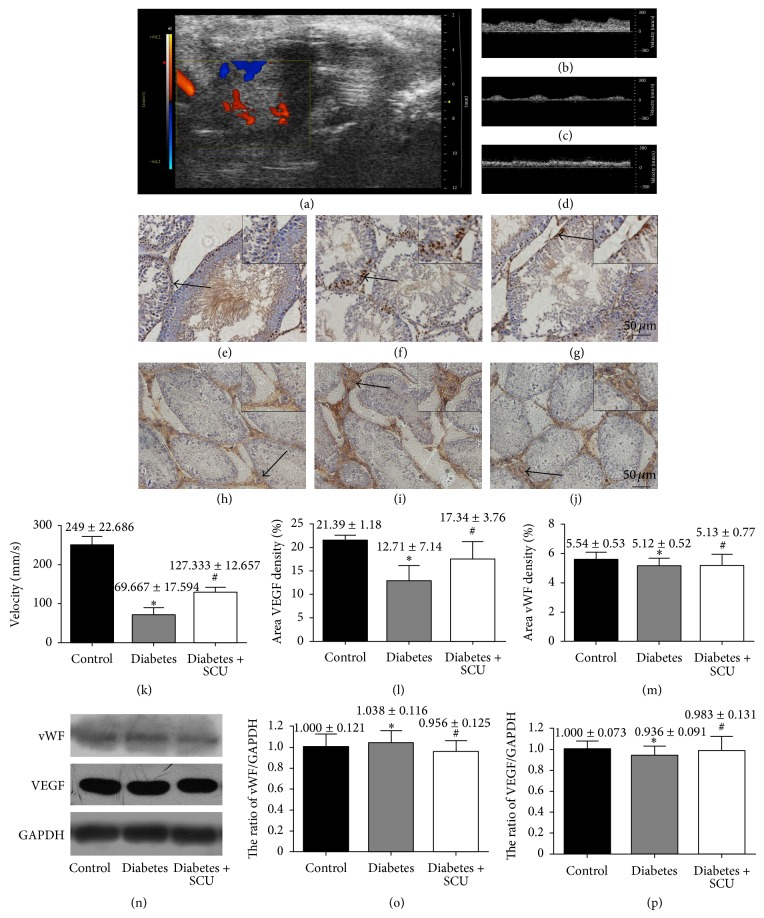
FSCU improve diabetes-induced microcirculatory changes. (a)–(d) Colour Doppler sonography analysis was performed in experimental animals. (a) Color Doppler ultrasound image from a diabetes + SCU rat testis showing localization of testicular blood vessel. (b)–(d) Spectral analysis of testicular blood flow rate in control, diabetes, and diabetes + SCU groups, respectively. (k) Quantitative evaluation of velocity. Data presented in (e) are means ± SEM (*n* = 8 per group; ^*^significantly different from control group (*P* ≤ 0.05), ^#^significantly different from diabetes group (*P* ≤ 0.05)). (e)–(j) The images of immunostaining of VEGF and vWF (arrows are used to indicate VEGF or vWF positive cells). (e) and (h) for control group, (f) and (i) for diabetes group, and (g) and (j) for diabetes + SCU group. DAB was used as background staining. VEGF or vWF density (% of area) was determined using Image-Pro Plus to calculate the total area of positive cells. Data in (l) and (m) are presented as means ± SEM, 3 fields per section and 5 sections from each testis, *n* = 8 rats for each group. (n) representative western blots show expression of vWF and VEGF. Bar graphs in (o) and (p) present quantitative difference in expression of vWF and VEGF. Data in (l) and (o) are presented as means ± SEM. ^*^Significantly different from control group (*P* ≤ 0.05), ^#^Significantly different from diabetes group (*P* ≤ 0.05). Data in (m) and (p) are presented as means ± SEM, *n* = 8 rats for each group. ^*^No significantly different from control group (*P* ≥ 0.05), ^#^No significantly different from diabetes group (*P* ≤ 0.05).

The effect of SCU on improving the testicular blood flow rate in diabetic rats was evaluated through analyzing the VTI. Color-coded Doppler ultrasound images of testicular blood were easily visualized (Figure 5(a)). VTI was decreased from 243.57 mm/s in control group to 56.87 mm/s in diabetes group (*P* < 0.05). In the diabetes + SCU group, VTI was obviously increased to 131.25 mm/s (*P* < 0.05) (Figure 5(k)). Cytoplasmic staining in the photographs indicated the expression of VEGF which is more prominent in control group, compared to diabetes group (Figures 5(e) and 5(f) with quantifications in Figure 5(l)). SCU treatment brought about increasing expression of VEGF in testes of diabetic rats, which were measured by immunohistochemical staining and western blot (Figures 5(g), 5(l), 5(n), and 5(o)) Immunohistochemical staining and western blot indicated that the testicular blood vessel density did not show any differences in these three groups (Figures 5(h), 5(i), 5(j), and 5(n), with quantifications in Figures 5(m) and 5(p)).

## 4. Discussion

DM, a state of chronic hyperglycemia, is a serious global disease with multiple organ and system dysfunction, including reproductive system [26]. Hyperglycemia-mediated apoptosis contributes to the damage of targeted organs [27, 28]. As the downstream effectors of hyperglycemia, oxidative stress and microcirculation impairment have been reported to play major role in triggering hyperglycemia-induced apoptosis. [17, 29]. Thus, the regimen that can both target oxidative stress and improve microcirculation is a promising strategy for prevention and treatment of diabetic complications, including reproductive disorder.

In this study, we evaluated the effect of SCU, a traditional herbal medicine, which has been shown to diminish oxidative stress and improve microcirculation in some diabetic complications [17], on hyperglycemia-induced apoptosis in testes of STZ-induced diabetic rats. Data showed that STZ-induced diabetic rats exhibited increased levels of oxidative stress detected by higher levels of ROS and lipid peroxidation, increased levels of apoptotic cells associated with upregulation of proapoptotic Bax and downregulation of antiapoptotic Bcl-2 in testes, and increased levels of microcirculation impairment demonstrated by decreased levels of blood flow velocity and VEGF, whereas SCU significantly reversed those hyperglycemia-induced actions. Our data, for the first time, suggest that SCU is a potent agent for prevention and treatment of diabetes-associated reproductive disorder.

STZ combined with HFD is a common way to induce T2DM in rat. STZ induces inflation and degeneration of Langerhans islets beta cells, leading to hyperglycemia [30]. Thus, hyperglycemia combined with body weight loss is the common marker for a success in induction of diabetes [31]. Data in Figures 1(a) and 1(b) show the significant body weight loss and increase in hyperglycemia level in diabetes group, indicating a successful induction of T2DM in our study. Data also show that SCU has effect on recovery of body weight loss but has no effect on glucose levels, demonstrating that SCU targets downstream effectors of hyperglycemia.

Body weight loss is a common symptom in STZ-induced diabetic rats, which is combined with organ weight loss. The reason for weight loss is that STZ possibly prevents the secretion of testosterone and growth hormone, resulting in disorder of anabolic activities [32]. Data in Table 1 show a significant decrease in the weights of testes and epididymis in diabetes group in comparison with control group, suggesting that the weight loss of reproductive organs may be one of the reasons to cause its dysfunction. Diabetes-induced weight loss of reproductive organs in male has been reported to be induced by oxidative stress, leading to atrophy of sex organs [33]. SCU totally reversed the weight loss of testes and epididymis to control level (Table 1,* P* ≤ 0.05), suggesting that downstream effector of hyperglycemia plays major role in the weight loss of reproductive organs in diabetic rat. In our study, ROS-induced apoptotic cell death and organ atrophy may be the major factor for the organ weight loss. It is noted that SCU totally reversed the weight loss of testes and epididymis but only partially blocked body weight loss (Figure 1(a)), suggesting that different mechanisms are applying for body weight loss and organ weight loss, respectively, in diabetes.

Growing evidence indicates that oxidative stress is increased in diabetes due to overproduction of ROS and decreased efficiency of antioxidant defences [34]. ROS, as an effector of hyperglycemia, occurs very early during diabetes development and plays major role in organ damage. ROS is well known to be associated with male infertility [35]. High level of ROS affects both sperm quality and function. The dominant lethal-type mutation in male, a genetic alteration in a gamete, can be induced under high oxidative stress, which kills the conceptus early in development [36]. As early as 5 days of STZ-induced rats, enhanced ROS and lipid peroxidation can be measured in testes. Male-mediated dominant lethal-type mutations, a genetic alteration in a gamete, can be induced under high oxidative stress, which kills the conceptus early in development [8]. ROS has been reported to induce apoptosis of testicular germ cells, leading to degeneration of testes and infertility in diabetic rats [37].

ROS-induced apoptosis is associated with ROS-induced mitochondrial DNA damage, leading to activation of DNA damage-mediated p53 signaling pathway, involving upregulation of proapoptotic Bax and downregulation of antiapoptotic Bcl-2 in testes of diabetic rats [5]. Our data showed that diabetes-induced obvious apoptotic morphological changes (Figure 4) and increased TUNEL positive cells (Figure 3) of testicular cells, as well as increased levels of proapoptotic Bax and decreased levels of antiapoptotic Bcl-2 in testes, were associated with increased levels of ROS and lipid peroxidation (Figures 2(a) and 2(b)) in diabetes group (Figure 2), suggesting that ROS induces apoptosis of testicular cells in our diabetic mode.

Recent study showed that sulforaphane, a controller of detoxification response, targeted antioxidative factor, Nrf2, to inhibit diabetes-induced cell apoptosis pathway in testes [38]. Actually, our research has a similar result that SCU, an antioxidant, blocks both hyperglycemia-induced ROS and apoptosis in testes further confirming that ROS contributes to hyperglycemia-induced apoptosis in testes.

Although researches have proven that sulforaphane has effect on suppressing oxidative stress and cell death, but little evidence indicates that it can improve diabetes-induced microcirculatory disturbance of testes [38, 39]. Microcirculatory disturbance is a common feature in diabetes, which plays critical role in organ damage [40]. Blood flow velocity is used to evaluate the change of bloodstream, which indicates the microcirculatory situation of organs. The lower level of blood flow velocity possibly results in lack of nutrients and deoxygenation of organs [41], in which apoptosis is triggered [42].

**Figure 6:**
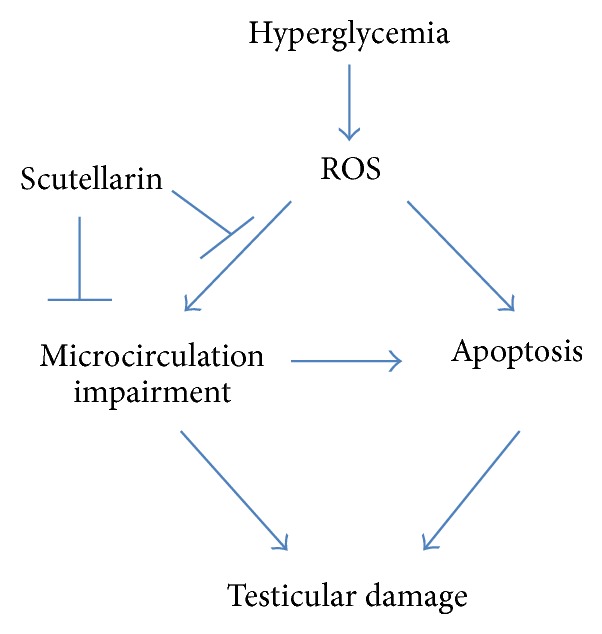
Schematic diagram for the mechanism by which SCU protects testicular damage in diabetic rat.

The mechanism underlying the blood flow abnormalities possibly relates to oxidative-induced endothelial dysfunction, leading to vasospasm and elevated blood viscosity [10]. There is no change of testicular blood vessel density in diabetic group (Figure 6); therefore the low expression of VEGF is manifested by endothelial dysfunction which can result in microcirculatory impairment [12]. Increased ROS triggers PARP/NF-*κ*B pathway, leading to vasospasm, enhanced coagulation of platelet and blood, and ischemic episodes [10, 43]. Thus, ROS induces apoptosis in diabetic organ via at least two mechanisms (Figure 6): (i) ROS directly triggers apoptosis via activation of ROS-mediated apoptotic signaling pathways, such as p53/Bcl-2 pathway, and (ii) ROS indirectly triggers apoptosis via microcirculatory disturbance, leading to lack of nutrients in organs, in which starving apoptotic pathway can be activated, such as mTOR pathway. Our data clearly showed lower levels of blood flow velocity in diabetic rats, suggesting that the testes lacked blood supply and the cells were in starving condition. It will be interesting to know if starving apoptotic pathway is involved in hyperglycemia-induced apoptosis in testes. Future study will address this issue.

SCU, a well-known polyphenolic flavonoid extracted from the Chinese traditional herb, Fleabane Compositae,* Erigeron breviscapus*, is the major effective ingredient of breviscapine, which is widely used in the treatment of cardiovascular diseases [19]. It is also being used in the treatment of cerebral ischemia and infantile acute viral myocarditis [44, 45]. SCU exhibits antioxidant property which has been studied for its protective effects on neuro [46], blood circulation [47], and nephropathy [18]. SCU has been reported to be effective in dilating blood vessels, increasing blood flow rate, improving microcirculation and hemodynamics, decreasing blood viscosity, and preventing platelet conglomeration [47, 48]. Due to its antioxidant property and its ability to improve microcirculation, SCU has been studied for prevention and treatment of diabetic complications, such as diabetes associated cardiovascular disease [19], diabetic nephropathy [18], and diabetic retinopathy [49]. However, whether SCU can prevent and treat diabetes associated productive disorder is elusive.

Present study, for the first time, demonstrated that SCU has the ability to block hyperglycemia-induced apoptosis, ROS, and microcirculation impairment in testes of STZ-induced diabetes rats. Since ROS are the major upstream event of microcirculation impairment and germ cells apoptosis in diabetes, in this study we could not clarify if SCU improves microcirculation via its antioxidant property, its ability to directly improve microcirculation, or both. In addition, we could not clarify if SCU blocks hyperglycemia-induced apoptosis via other mechanisms than ROS, such as its direct effect on improving microcirculation. Further study is needed to better understand the mechanisms of SCU's actions and try to answer the question: is SCU better than other antioxidants in improving diabetes associated reproductive disorder? Recent study showed that sulforaphane also can protect against T2DM-induced testicular apoptosis [39]. In the future, we can compare the effect between sulforaphane and SCU in protecting against T2DM-induced reproductive problems.

## 5. Conclusion

Taken together, our data suggest that SCU possesses the potential to reverse diabetes associated male reproductive disorder, providing first in vivo evidence to support further clinic study to use SCU as a nontoxic drug to prevent and treat diabetes-mediated reproductive disorder. Data also suggest that targeting ROS is one of the mechanisms whereby SCU blocks hyperglycemia diabetes-induced apoptosis of germ cells and improves hyperglycemia-induced microcirculation impairment in testes. Further clinic study is needed to verify the SCU's action.

## Supplementary Material

Supplemental table 1A: Mean values of body weights of three experimental groups at every week. Data are presented as mean ± SEM. Weights of all groups are increased from week 1 to week 8. Weights of diabetes group and Diabetes + SCU group show a decreased tendency from week 9, while control group is keep on growing.
Supplemental table 1B: Mean values of blood glucose of three experimental groups at 8-16week. Data are presented as mean ± SEM. Control group show a normal level of plasma glucose, but it in diabetes group and diabetes + SCU group are higher than diabetic level.
Supplemental table 2: Comparison of serum TG, TC, LDL and HDL in three experimental groups. Data are presented as mean ± SEM. There are no differences between those groups.

## Figures and Tables

**Table 1:** Testes weights and epididymal weights of experimental animals.

Weight (g)	Control group	Diabetic group	Diabetes + SCU group
Testes	1.97 ± 0.30	1.32 ± 0.13^*^	1.70 ± 0.18^†^
Epididymis	0.56 ± 0.10	0.31 ± 0.09^*^	0.44 ± 0.08^†^

^*^Significantly different from control group (*P* ≤ 0.05).

^†^Significantly different from diabetes group (*P* ≤ 0.05).

Values are expressed as mean ± standard error of the mean (SEM).
