# Supplementary material for: Protective Effects of Scutellarin on Type II Diabetes Mellitus-Induced Testicular Damages Related to Reactive Oxygen Species/Bcl-2/Bax and Reactive Oxygen Species/Microcirculation/Staving Pathway in Diabetic Rat
Source: J Diabetes Res. 2015 Mar 12;2015:252530. doi: 10.1155/2015/252530 (PMC4377542; doi:10.1155/2015/252530)
Supplement: Supplementary file 1 — Supplemental table 1A: Mean values of body weights of three experimental groups at every week. Data are presented as mean ± SEM. Weights of all groups are increased from week 1 to week 8. Weights of diabetes group and Diabetes + SCU group show a decreased tendency from week 9, while control group is keep on growing. Supplemental table 1B: Mean values of blood glucose of three experimental groups at 8-16week. Data are presented as mean ± SEM. Control group show a normal level of plasma glucose, but it in diabetes group and diabetes + SCU group are higher than diabetic level. Supplemental table 2: Comparison of serum TG, TC, LDL and HDL in three experimental groups. Data are presented as mean ± SEM. There are no differences between those groups. [file 252530.f1.pdf]

Supplemental table

Supplemental table 1A. Mean values of body weights of three experimental group at every week. Data are presented as mean ± SEM.

	Body weight(g)		
	Control	Diabetes	Diabetes+SCU
Week1	240.67±7.45	243.00±3.61	246.17±15.16
Week2	245.50±12.08	246.50±9.25	249.67±13.58
Week3	245.34±15.38	258.50±5.44	260.67±14.46
Week4	249.17±14.45	266.67±3.68	265.34±13.47
Week5	257.17±18.76	275.34±6.24	291.50±17.10
Week6	257.34±17.19	282.37±7.52	302.17±17.88
Week7	254.17±15.44	291.84±12.54	314.34±18.46
Week8	269.00±26.79	308.00±14.88	312.34±16.18
Week9	283.67±21.79	309.34±20.52	285.32±20.80
Week10	295.67±23.41	295.00±21.15	278.50±19.25
Week11	320.00±30.13	278.33±21.37	269.50±21.82
Week12	306.34±22.11	262.50±22.82	251.84±27.75
Week13	309.17±28.12	239.50±23.08	242.50±26.29
Week14	320.67±25.55	224.34±22.65	242.50±29.63
Week15	335.17±31.02	208.34±31.01	224.83±22.46
Week16	347.67±33.76	201.50±32.87	234.34±21.97

Supplemental table 1B. Mean values of blood glucose of three experimental group at 8-16week. Data are presented as mean ± SEM.

	Plasma glucose (mmol/L)		
	Control	Diabetes	Diabetes+SCU
Week8	6.73±0.61	5.53±0.70	5.98±0.66
Week9	6.57±0.19	28.05±0.73	26.38±0.39
Week10	6.71±0.58	27.98±0.74	26.50±0.60
Week11	6.87±0.46	28.08±0.75	26.32±0.41
Week12	7.03±0.47	28.02±0.75	26.53±0.89
Week13	7.13±0.26	28.15±0.89	26.34±0.808
Week14	7.16±0.40	28.00±0.73	26.34±0.41
Week15	7.10±0.21	27.95±0.64	26.54±0.61
Week16	6.90±0.22	27.92±0.73	26.48±0.43

Supplemental table 2. Comparison of serum TG, TC, LDL)and high-density lipoprotein (HDL) in three experimental groups. Data are presented as mean ± SEM.

	Control	Diabetes	Diabetes+SCU
TC (mmol/L)	0.92±0.13	1.16±0.19	1.10±0.23
TG (mmol/L)	0.99±0.17	1.13±0.25	0.97±0.41
HDL-C (mmol/L)	1.21±0.26	1.47±0.34	1.39±0.38
LDL-C (mmol/L)	0.67±0.13	0.74±0.14	0.75±0.26
